# Tensile Behaviour of Double- and Triple-Adhesive Single Lap Joints Made with Spot Epoxy and Double-Sided Adhesive Tape

**DOI:** 10.3390/ma15217855

**Published:** 2022-11-07

**Authors:** Przemysław Golewski

**Affiliations:** Department of Solid Mechanics, Faculty of Civil Engineering and Architecture, Lublin University of Technology, Nadbystrzycka 38, 20-618 Lublin, Poland; p.golewski@pollub.pl

**Keywords:** dual-adhesive, triple-adhesive, double-sided adhesive tape, DIC, SLJ

## Abstract

Dual adhesives are mainly used to increase the strength of single lap joints (SLJs) by reducing the stress concentration at its ends. However, they can also be used to design the characteristics of the joint so that its operation and failure occur in several stages. This paper presents the results of uniaxial tensile tests for dual-adhesive and triple-adhesive SLJs. The adherends were made of aluminum and glass fiber-reinforced polymer (GFRP) composite. For dual-adhesive SLJs, 10 epoxies and 1.6 mm thick double-sided adhesive tape were used. The stiffest (Epidian 53 (100 g) + “PAC” hardener (80 g)) and most elastic (Scotch-Weld 2216 B/A Translucent) joints were determined, which were then used in a triple-adhesive joint with the same double-sided adhesive tape. Circular holes of different diameters from 8 mm to 20 mm were made in the double-sided adhesive tape, which were filled with liquid epoxy adhesive by injection after the adherends were joined. By using the double-sided adhesive tape, the geometry of the epoxy joints was perfect, free of spews, and had a constant thickness. The effect of the spot epoxy joint diameters and the arrangement of stiff and elastic joints in the SLJs were analyzed using digital image correlation (DIC).

## 1. Introduction

The continuous effort to reduce the weight of structures while increasing their strength has led to the increasing use of polymer–matrix composites (PMC). Despite their many advantages, such as a favorable weight-to-strength ratio, resistance to weathering and the ability to design complex shapes, PMC composites with an epoxy matrix are more difficult to join compared to metals. Metal structures can use techniques such as welding [1], spot welding [2], friction stir welding [3], and clinching [4]. In contrast, epoxy–matrix PMC composites are insulators, do not deform plastically, and suffer thermal degradation when exposed to high temperatures. Therefore, the range of joining techniques available for use with such composites is limited. The universal joining technique is mechanical: removable in the form of bolts [5] and non-removable in the form of rivets [6,7]. Both of these techniques can be used to join composites to other materials, but mechanical joints usually require a hole, and stress concentrations occur, which is dangerous with the brittle linear characteristics of the PMC material [8]. An alternative is adhesive joints [9,10,11], which do not require interference with the structure of the parts to be joined. Their characteristic feature is that the stress concentrates at the ends of the lap [12], which makes this type of joint sensitive to the technology of their manufacture. In order to increase their strength, a number of techniques are used, such as edge chamfering [13], surface modification [14], modification of the spew geometry [15,16], stepped reduction of the thickness of the parts to be joined to eliminate eccentricity [17,18], and a different length-to-width ratio of the joint [19]. These types of techniques would often be difficult to apply in industrial environments due to the cost of additional machining. Therefore, another technique may be the use of hybrid connections [20,21,22,23,24] in which both a mechanical joint (rivet, bolt, weld) and an adhesive joint are used. Due to differences in stiffness and the occurrence of plastic deformation, the operation of such hybrids can be two-stage, which increases the safety of the structure, for example, if one of the joints is damaged. However, the problem of significant interference with the structure of the materials being joined still remains. Therefore, one of the varieties of hybrid joints is dual-adhesive or mixed adhesive joints, which were first proposed in 1966 by Raphael [25]. This solution is based on the use of layers of different adhesives in one joint. These layers have different stiffnesses. The less rigid layer is placed at the ends of the lap where the greatest deformation of the adhesive occurs. The more rigid layer is inside the overlap, where the deformation is much smaller. Dual adhesive joints are used to increase the strength of the connection. However, they can also be used to design safe, multi-stage joints and to design operating characteristics. Current work in the field of dual-adhesive joints is being carried out towards strengthening tensile lap joints [26] and flexural beams [27]. In the work [28], the authors split lap length in the ratio of 1/3 elastic adhesive, 2/3 rigid adhesive, and the effect of the surface roughness and the lap length were also analyzed. Single adhesive joints were also studied, but the combination of two adhesives produced an increase in strength for all the analysed models. Dual adhesive joints are also subjected to the effects of low and high temperatures [29], and ageing in humid environments [30] and under dynamic loads [31,32], as they have significant potential for application in the automotive, aerospace, and aviation industries, replacing joints with only one adhesive layer. The problem in dual-adhesive joints featuring two liquid adhesives is the method of separating them. Silicone [33,34] and nylon fiber [35] separators are used for this purpose, or alternatively they are allowed to mix with each other at the interface [36]. On the other hand, the authors in the works [37,38] proposed using double-sided adhesive tape as a separator. Such solutions mean that after using only one type of liquid epoxy adhesive, the joint will have two-stage characteristics. In the first stage, the rigid epoxy joint is damaged, while in the next stage, the load is transferred by the double-sided adhesive tape, making the total energy of the damage very high. These types of joints can also be called safety joints. However, until now, no one has used this technique in the configuration: double-sided adhesive tape + 2 types of liquid epoxy adhesives.

The research was divided into two stages. The first stage involved dual-adhesive joints, where an adhesive tape was used in addition to an epoxy joint. The goal of the first stage was to test 10 types of resin + hardener configurations to determine which had the lowest and highest stiffness. This stage was followed by triple-adhesive specimens. In both cases, two materials were combined: 1 mm thick GFRP laminate and 2 mm thick aluminum. The double-sided adhesive tape used in both cases had a thickness of 1.6 mm. Five samples per batch were used, hence the total number of samples was 100.

## 2. Materials and Methods

In this study, 10 epoxy compositions were used in the form of commercial adhesives and resins along with hardeners.

Two commercial epoxy adhesives were used:

- Distal, produced by Libella, and
- Scotch-Weld 2216 B/A Translucent, produced by 3M.

Other compositions of epoxy adhesives resulted from the use of two resins: Epidian 5 and Epidian 53, as well as two hardeners: PAC and Z1, produced by Ciech, Sarzyna.

PAC is a brown-colored liquid polyamide hardener with relatively low reactivity. The weight ratio of PAC hardener to resin can be varied over a wide range to regulate the reaction rate and properties of the cured material. Compositions richer in PAC hardener are more flexible and resistant to impact, but less hard and less resistant to elevated temperatures. Therefore, this study examined its effect in the range from 60 g to 100 g per 100 g of Epidian 5 resin and from 50 g to 80 g per 100 g of Epidian 53 resin.

Z1 is a thin, colorless liquid. Thanks to its low viscosity, it is used for making epoxy composites by the following processes: hand lamination, vacuum lamination, and repair. The Z1 hardener is very efficient and forms a fairly stiff composite with improved temperature resistance. The addition of Z1 hardener to Epidian 5 and Epidian 53 resins is strictly defined and is, respectively, 10 g and 12 g per 100 g of resin.

Data concerning work life, time to handling strength, and curing time for all epoxy compositions are shown in Table 1.

The application of the epoxy adhesives was simplified by the use of double-sided adhesive tape. In this case, 3M VHB 5962 tape with a thickness of 1.6 mm was used.

**Figure 1:**
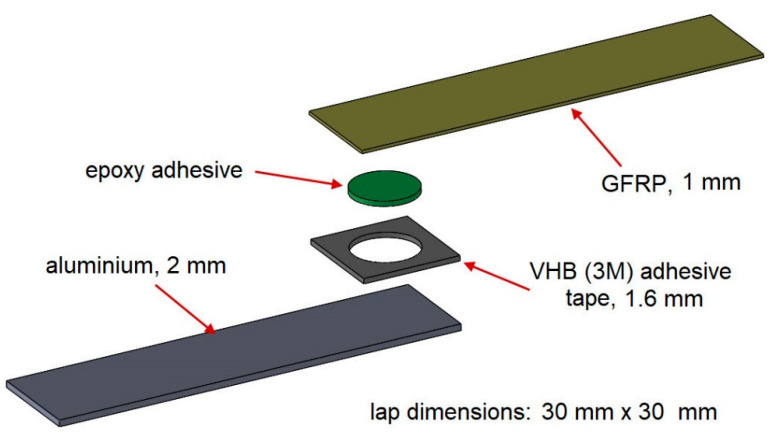
Structure of the dual-adhesive sample for the first stage of the study.

The research was divided into two stages. In the first stage of the study, the dimensions of the lap were 30 mm × 30 mm (Figure 1). The diameter of the epoxy joint was 20 mm. By using double-sided adhesive tape, high repeatability of the geometry could be achieved.

The aim of the first stage was to analyze 10 types of joints differing in the composition of the spot epoxy bond with respect to the maximum strength and stiffness. The following epoxy compositions were used in the samples:

1. Distal Classic (resin 100 g + hardener 74 g),
2. 3M Scotch-Weld 2216 B/A Translucent (resin 100 g + hardener 100 g),
3. Epidian 5 (100 g) + hardener “Z1” (10 g),
4. Epidian 53 (100 g) + hardener “Z1” (12 g),
5. Epidian 5 (100 g) + hardener “PAC” (60 g),
6. Epidian 5 (100 g) + hardener “PAC” (80 g),
7. Epidian 5 (100 g) + hardener “PAC” (100 g),
8. Epidian 53 (100 g) + hardener ”PAC” (50 g),
9. Epidian 53 (100 g) + hardener ”PAC” (65 g),
10. Epidian 53 (100 g) + hardener ”PAC” (80 g).

There were 5 samples in each batch. Hence, the total number of samples in the first stage of testing was 50. As a result of the analysis, the two most extreme adhesive compositions were determined: the stiffest and the most elastic, which were then used in the second stage of testing.

**Figure 2:**
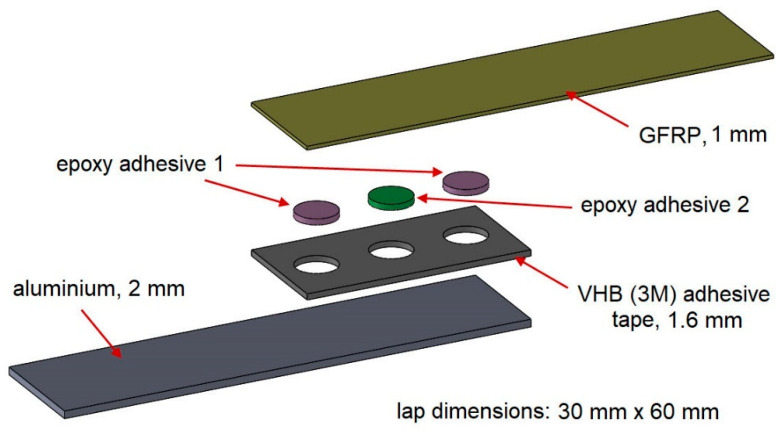
Structure of the triple-adhesive sample in the second stage of study.

In the second stage of the study, the lap was much more complicated. The length of the lap was 60 mm, with the same width as in the first stage. There were three spot bonds in each lap (Figure 2):

− one central spot bond, whose diameters were: 8 mm, 11 mm, 14 mm, 16 mm, and 20 mm,
− two spot bonds placed at the ends of the lap with the same diameter of 11 mm in each batch.

**Figure 3:**
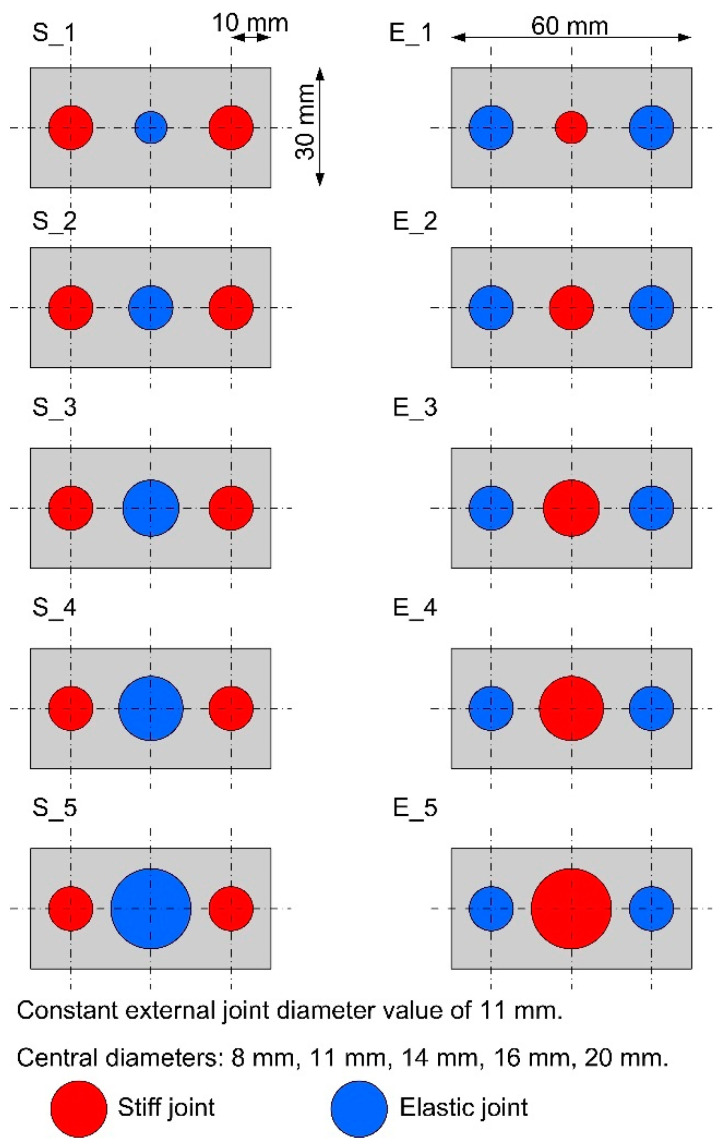
Schemes of lap construction in triple-adhesive samples.

Making this type of spot bond was possible through the use of 3M VHB 5962 double-sided adhesive tape with a thickness of 1.6 mm. The samples were divided into two groups: “S” and “E”. In the “S” type specimens, the outer joints were stiff, while the central joint was elastic. In the “E” type specimens, the situation was the opposite. A diagram of all types of specimens in the second stage of testing is shown in Figure 3. There were 5 specimens in each batch, so the total number of specimens in the second stage of testing was 50.

The purpose of such changes in geometry and swapping the location of the stiff and elastic bonds was to analyze the effect on the SLJs characteristics, maximum force, and failure energy.

In both the first and second stages of the study, the adherends were made of two different materials: 2 mm-thick AW-6060 aluminum and 1 mm-thick GFRP composite, EPGC 201 from Izo-Erg, Gliwice, Poland. The surfaces were cleaned with Loctite 7061 before bonding.

**Figure 4:**
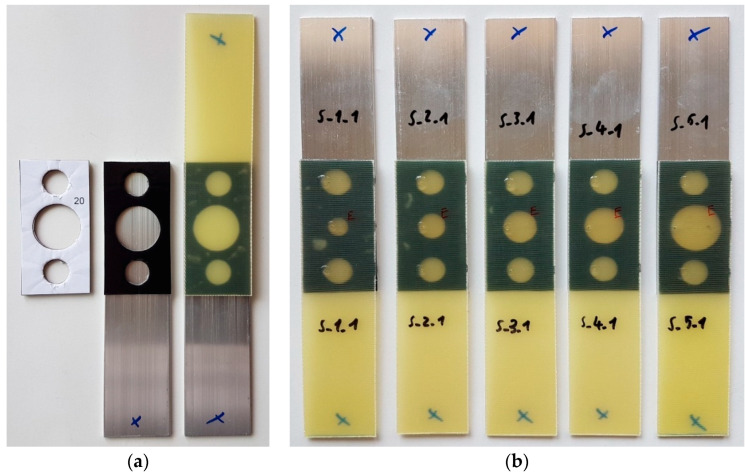
Triple-adhesive samples: (**a**) stages of fabrication; (**b**) completed samples.

Manufacturing accurate geometries in both the first and second stages of the study was made possible by the use of double-sided adhesive tape, in which holes were cut with die-cutters before the tape was applied to the substrate. After the holes were made, the tape was then applied to one of the adherends. After removing the protective foil, the second adherend was applied. In this way, the samples were ready for the epoxy adhesive to be applied, which was done by injection into the empty spaces that were formed between the composite and aluminum. Complete samples from the first “S” batches can be seen in Figure 4.

The samples were cured at room temperature for one week. After this time, they were subjected to static uniaxial tensile tests on an MTS 100 kN testing machine. According to ASTM D1002-10(2019), the loading rate was 1.27 mm/min. The results from the testing machine were processed in the Diadem software (2019).

An Aramis digital image correlation (DIC) system was used to observe strains on the surfaces of the laps. The results from the Aramis system were processing in the GOM Correlate software (2020).

## 3. Results and Discussion

The research was divided into two stages, and 50 lap specimens were tested in each stage. In the first stage of testing, the behavior of 10 batches of dual-adhesive joints was determined to identify the two extreme types of joints, taking into account their stiffness.

In the second stage of testing, the behavior of 10 batches of joints differing in both the geometry and location of stiff and elastic joints was determined. The DIC system was also used to observe deformations in this stage.

### 3.1. Results for the First Stage of the Study

**Figure 5:**
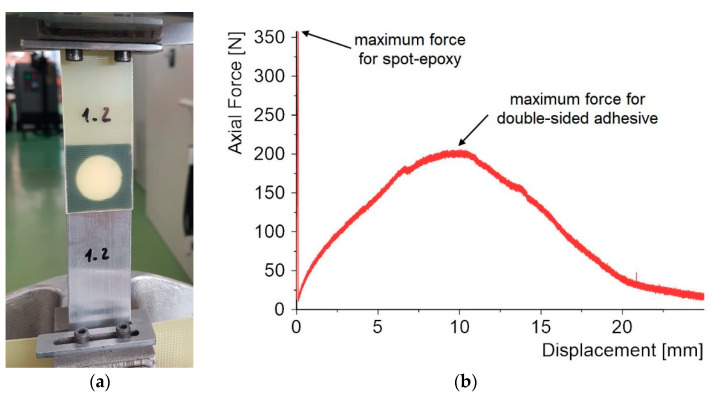
Static tensile test for dual-adhesive type specimens: (**a**) specimen during testing, (**b**) force–displacement diagram (specimen 1_2).

Dual-adhesive specimens with one epoxy bond and a double-sided adhesive tape were used in the first stage of the work. These are materials that differ in both shear strength and stiffness. The failure of this type of joint proceeds in two stages. First, the rigid epoxy joint is damaged. This process occurs quite quickly for displacement in the range of 0.2–1 mm. In the next stage, the double-sided adhesive tape begins to work, which is damaged over a relatively long displacement, as can be seen in the graph in Figure 5b. However, for this part of the study, the first stage of the work and the comparison of 10 epoxy adhesives are the most interesting parts.

**Figure 6:**
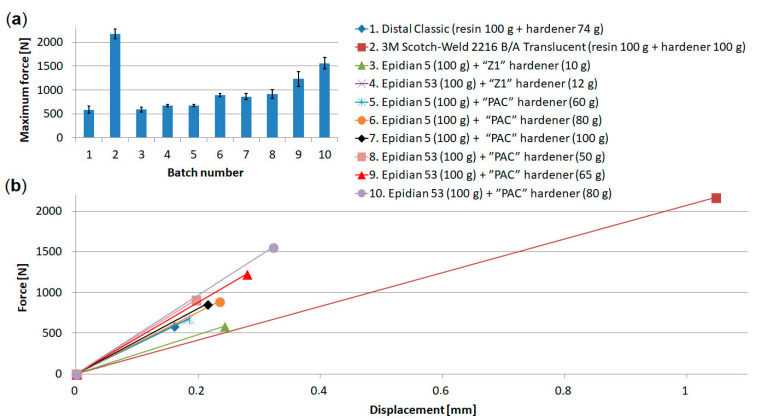
Summary of results of the first stage of joint operation: (**a**) summary of maximum forces, (**b**) comparison of stiffness for epoxy joints.

Figure 6 shows the results for the first stage of joint operation.

Figure 6a shows the maximum force values for 10 batches of dual-adhesive specimens. The maximum force value in each case was obtained for the epoxy joint. The highest maximum force (2172 N) was achieved for joints using Scotch-Weld 2216 adhesive (batch 2). The lowest value (588 N) was achieved by joints using Distal Classic adhesive (batch 1) and Epidian 5 (100 g) + “Z1” hardener (10 g) (batch 3).

Figure 6b was created by connecting point (0, 0) with the points corresponding to the maximum force and displacement, i.e., the failure of the epoxy joint, whose characteristics are linear. The type of resin or adhesive used, as well as the proportion of hardener used, have a significant influence on the strength and stiffness. The Scotch-Weld adhesive had the lowest straight angle and the lowest stiffness. The adhesive with Epidian 53 resin (100 g) and PAC hardener (80 g) had the highest stiffness. These two compositions were selected for further testing, for the formation of triple-adhesive joints.

### 3.2. Results for the Second Stage of the Study

The adhesive joint is stressed to varying levels during the tensile test of the SLJ. The adhesive material closer to the end of the lap deforms to a greater extent than in the axis of the lap. Therefore, the method of placement of the stiff joint and the elastic joint is of great importance in this case. In classic dual-adhesive joints, the elastic joint is always placed in the end zone of the lap, while the stiff joint is placed closer to the axis of the lap. This arrangement helps increase the strength of the whole connection. However, the question arises: what will the force–displacement diagrams look like when the two adhesives are swapped in place? In addition, the characteristics can be formed by different percentages of the two adhesives: stiff and elastic. Therefore, different diameters of the central joint were considered.

**Figure 7:**
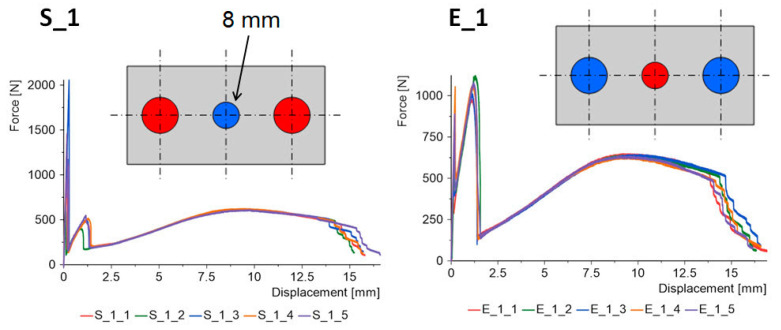
Triple-adhesive samples, batch 1.

**Figure 8:**
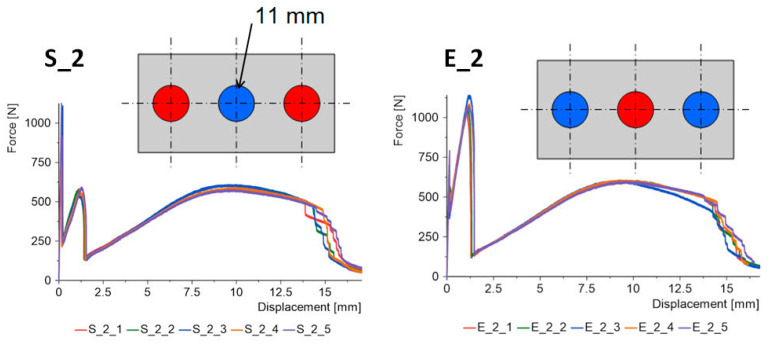
Triple-adhesive samples, batch 2.

**Figure 9:**
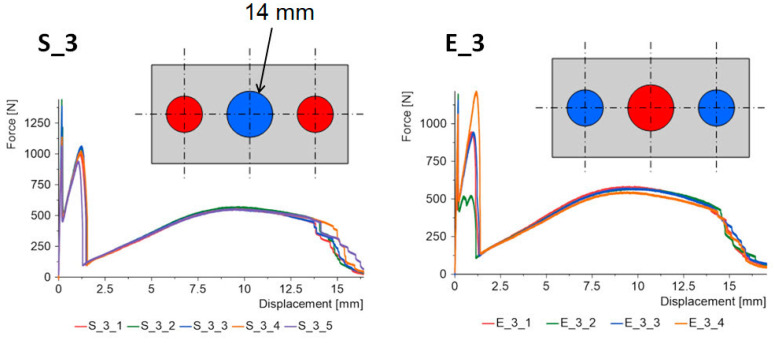
Triple-adhesive samples, batch 3.

**Figure 10:**
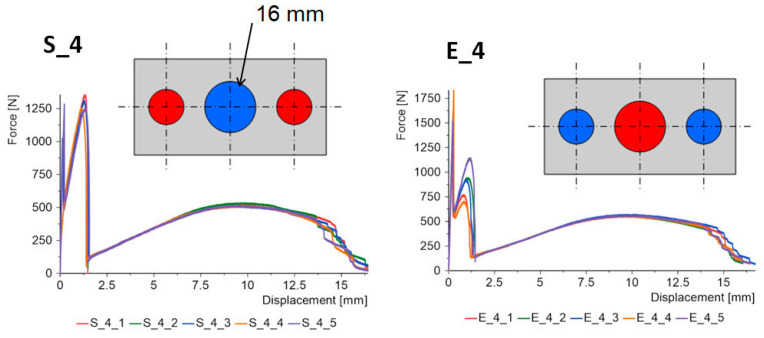
Triple-adhesive samples, batch 4.

**Figure 11:**
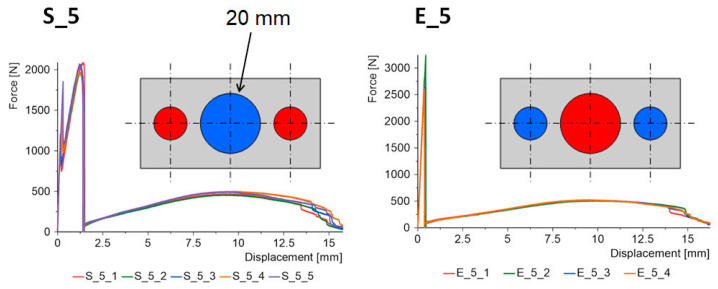
Triple-adhesive samples, batch 5.

The force–displacement graphs for the “S” and “E” groups for the same geometries are summarized in Figure 7, Figure 8, Figure 9, Figure 10 and Figure 11. The results for the first batches with the smallest central diameter of 8 mm are shown in Figure 7. With two rigid joints on the outside, we can double the strength compared to the “E” specimens. However, the “E” specimens reach a force of about 1 kN twice, which increases the safety of the joint.

By increasing the diameter of the central joint to 11 mm (Figure 8) in the “E” type specimens there is a decrease in the maximum force, while the force for the second stage is slightly increased. In the “S” type specimens, we encounter the phenomenon that the maximum force is reached in the second stage of the work.

Another increase in the diameter of the central joint to 14 mm (Figure 9) greatly benefits the “S” type specimens. The strength for the first and second stages increases, but the value for the first stage still dominates. In the “E” type specimens, large variations in the results for the second stage were noted, which could be due to possible errors in the preparation of the samples.

The optimal solution in terms of safety was obtained for the “S” type specimens with a central joint diameter of 16 mm (Figure 10). It comes down to the equalization of forces for the first and second stages. In the “E” type specimens, the reverse process occurs. The force value for the first stage increases, while it decreases for the second stage.

The last batches had a central joint diameter of 20 mm (Figure 11). This increase again benefits the “S” type specimens. In this case, the maximum forces for the second stage were obtained at 2 kN. A very interesting phenomenon occurs for the “E” type specimens. One of the working stages of the epoxy joint disappears, which is a negative outcome. Instead, the maximum force is obtained in relation to all the samples tested, with an average for the 5 samples of 2.8 kN.

So far, nothing has been mentioned about the third stage, which concerns the work of the double-sided adhesive tape. In this stage, nothing special happens; similar force values are obtained for all samples.

A common feature of each batch is the occurrence of a third stage, which is related to the work of the double-sided adhesive tape. This stage begins after the damage of all epoxy joints, which occurs for a displacement of about 1.3 mm and continues until a displacement of 15 mm, which corresponds to half the length of the lap. This stage is characterized by a maximum force in the range of 480 N–620 N.

**Figure 12:**
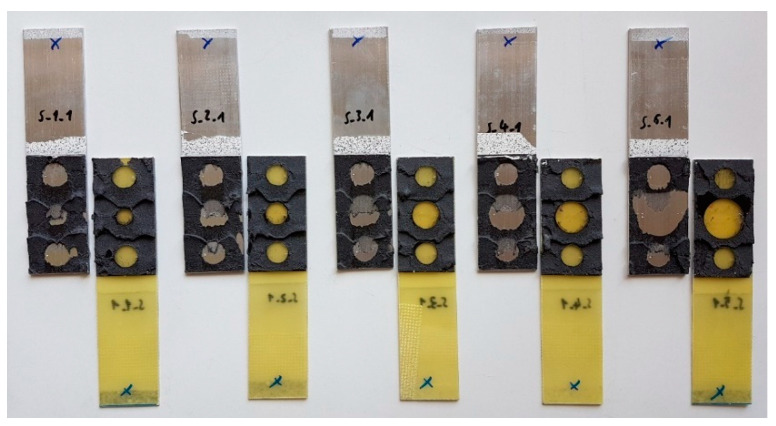
Triple-adhesive group “S” samples after damage.

**Figure 13:**
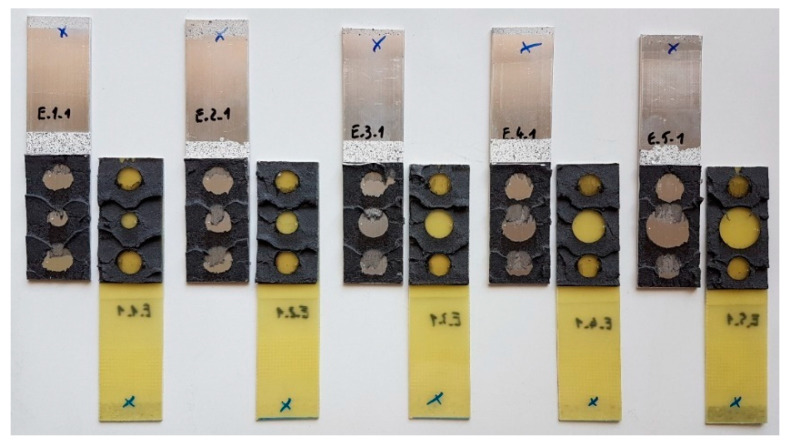
Triple-adhesive group “E” samples after damage.

Figure 12 and Figure 13 show images of one specimen from each batch after the uniaxial tensile testing. For both the “S” and “E” group specimens, there is a cohesive damage pattern for double-sided VHB tape. On the other hand, the damage of the spot epoxy bonds is adhesive in nature. The epoxy bonds remain on the composite adherend, which indicates much greater adhesion to the polymer material than to the aluminum. None of the adherends were damaged by exceeding the tensile strength during testing.

**Figure 14:**
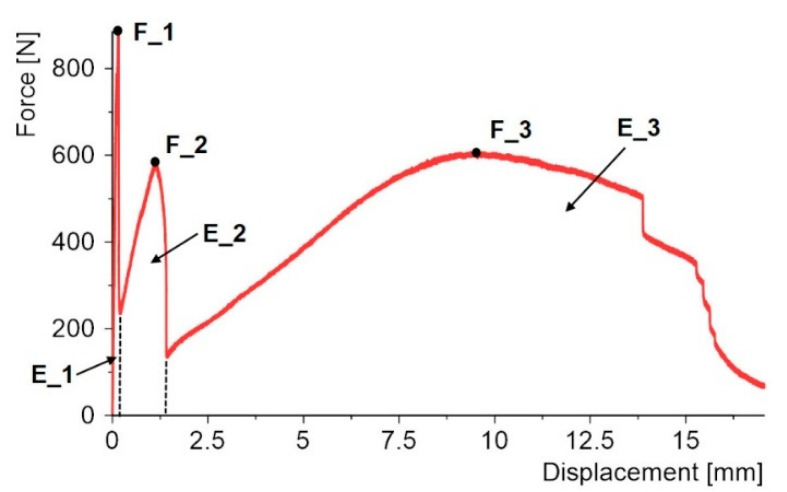
Determination of maximum forces and energies for triple-adhesive specimens.

The use of two different epoxy bonds and a double-sided adhesive tape caused the SLJ operation to proceed in three stages, as shown in Figure 14.

In the first stage, the load is mainly carried by the stiffer epoxy bond; after its damage, the force does not drop sharply to zero, but the load is taken up by the epoxy bond or bonds with less stiffness. Only after all the epoxy bonds have been damaged is there another drop in force, and the load is carried by the last joint in the form of the VHB tape.

In each of the above-mentioned stages, the maximum value of the force can be distinguished: F_1, F_2, and F_3. The field under the force–displacement diagram corresponds to the energy required to damage each joint and can be divided as indicated in Figure 14: E_1, E_2, and E_3.

**Figure 15:**
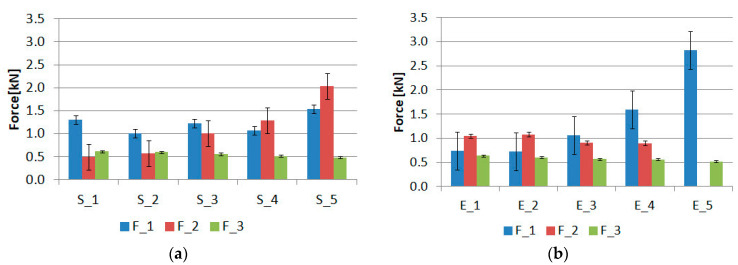
Summary of maximum forces for samples of group: (**a**) “S” and (**b**) “E”.

Figure 15 collects the average values of the forces for each of the batches, along with a classification of the three stages of operation of the joints. The exception is the “E_5” batch, where the second stage associated with elastic bond work is absent.

Analyzing the samples of the “S” group, which had two external rigid bonds, the following cases can occur depending on the diameter of the internal elastic bond, shown in Table 2.

Thus, there is a strong possibility of shaping the characteristics by making small changes in the value of the elastic bond area. Considering the two extreme cases of S_1 and S_5, when the internal diameter is changed from 8 mm to 20 mm, an increase of 18.5% is obtained for the F_1 force, while an increase of 314% is obtained for the F_2 force.

The value of the F_3 force is reduced by 22.5% due to a decrease in the area of the VHB tape.

Analyzing the samples of the “E” group, which had two external flexible welds, it is also possible to distinguish three stages of operation, except for the E_5 batch in which the second stage is absent. Depending on the internal diameter of the stiff bond, the following cases can occur, shown in Table 3.

Thus, using the reverse application of epoxy adhesives, we get different results in almost every batch. The exceptions are the batches S_3 and E_3, for which the F_1, F_2, and F_3 forces contribute, respectively:

- S_3-(F_1 = 1.23 kN, F_2 = 1.02 kN, F_3 = 0.56 kN)
- E_3-(F_1 = 1.06 kN, F_2 = 0.91 kN, F_3 = 0.57 kN)

For these two batches, there is the same order of forces, F_1 > F_2 > F_3, with the values for the forces in the same stages differing by 16%, 12%, and 1.75%. Therefore, it is possible to formulate the hypothesis for the presented triple-adhesive model that there is an arrangement of the lap geometry in which swapping the places of the stiff and elastic bonds does not cause changes in the values of the forces in the work stages. However, this issue requires further research.

**Figure 16:**
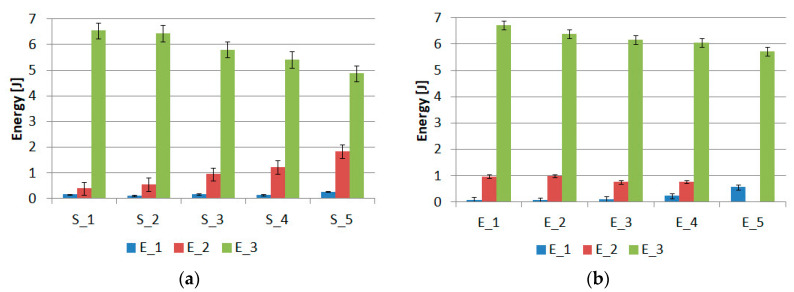
Summary of damage energy for samples of group: (**a**) “S” and (**b**) “E”.

The area under the force–displacement diagram indicates the energy required to damage the individual joints. A summary of the average values for the individual “S” and “E” batches is shown in Figure 16.

In each case, the energy level associated with the damage of the double-sided adhesive tape dominates. In both groups of “S” and “E” specimens, there is also the effect of decreasing the energy associated with the work of the double-sided tape as the diameter of the internal bond increases. Changing the internal bond from 8 mm to 20 mm results in an energy decrease of 25.5% for the “S” group samples and 14.7% for the “E” group samples. The smaller decrease for the “E” group samples is due to the fact that there is an internal stiff bond, which relieves the double-sided tape much more. The lowest energy, regardless of the type of sample, is characterized by the first stage of joint work. The energy achieved is in the range of 0.08 J–0.57 J. The energy for the second stage is in the range of 0.4 J–1.84 J. The larger effect on the energy in the second stage of the change in diameter can be observed for the samples of the “S” group.

**Figure 17:**
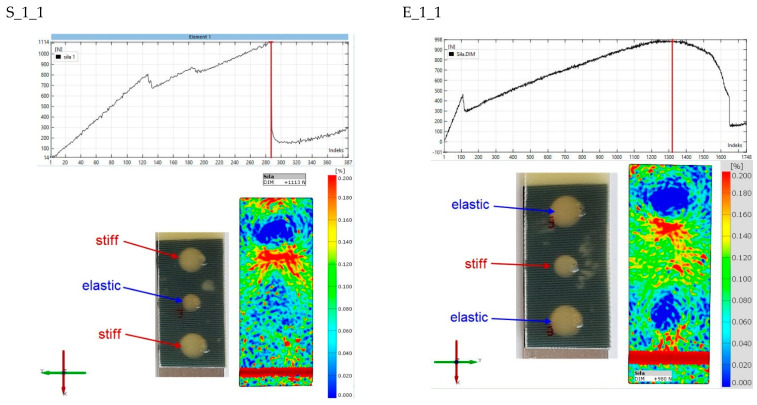
Comparison of principal strain maps for “S” and “E” batch 1 specimens for maximum force in the second stage of operation.

**Figure 18:**
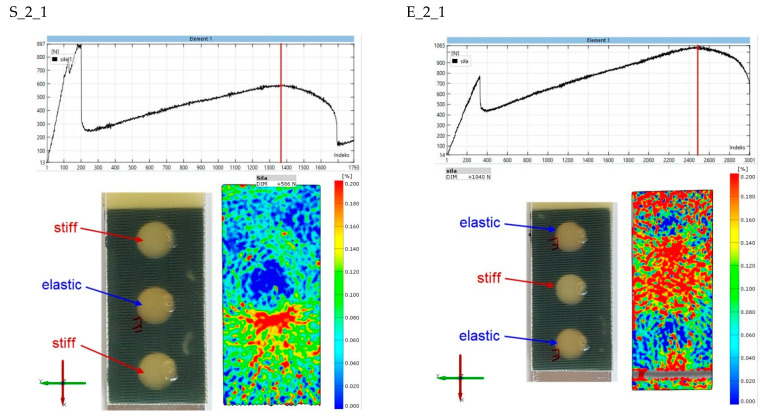
Comparison of principal strain maps for “S” and “E” batch 2 specimens for maximum force in the second stage of operation.

**Figure 19:**
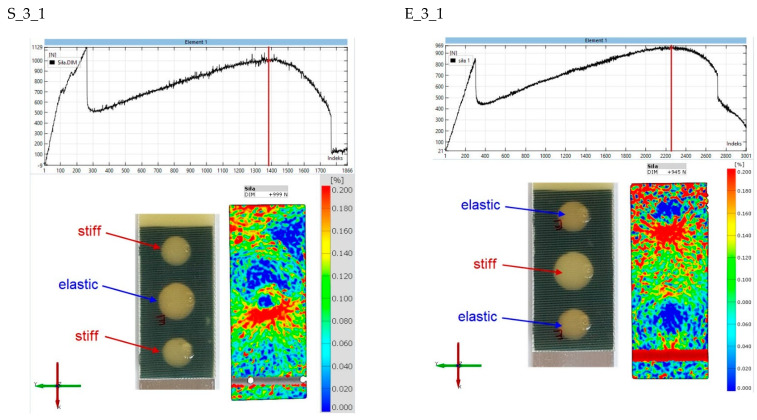
Comparison of principal strain maps for “S” and “E” batch 3 specimens for maximum force in the second stage of operation.

**Figure 20:**
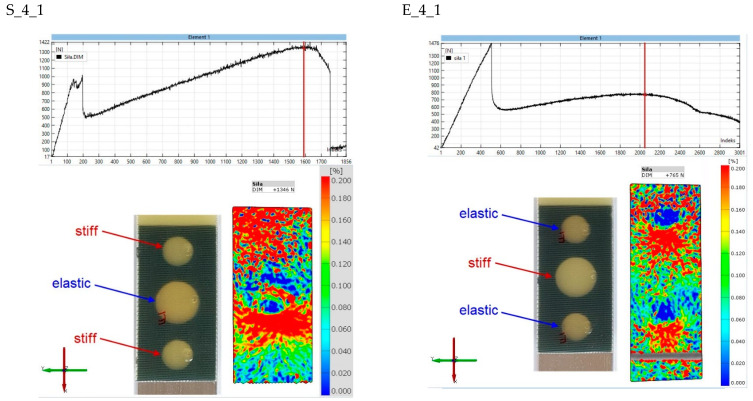
Comparison of principal strain maps for “S” and “E” batch 4 specimens for maximum force in the second stage of operation.

**Figure 21:**
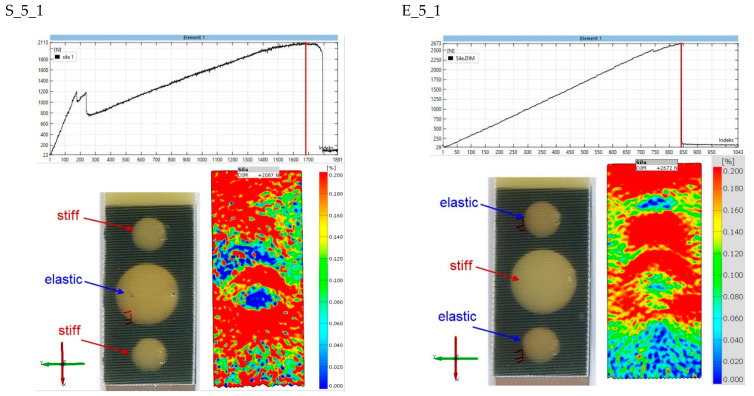
Comparison of principal strain maps for “S” and “E” batch 5 specimens for maximum force in the second stage of operation.

The final stage of the analysis concerns strains on the surface of the composite lap. The purpose of the observation was to determine the strains in the second stages of the joint operation. Figure 17, Figure 18, Figure 19, Figure 20 and Figure 21 show the results for one sample from each “S” and “E” series and for only the second stage of the epoxy joint operation.

The principal strains are shown for all samples. In addition, the scale range is limited from 0 to 0.2. Hence, the blue fields indicate small or zero strains, while the red fields indicate strains close to 0.2 or greater. Thus, analysis using the Aramis system, which uses digital image correlation, can provide a tool for observing the operation of spot adhesion joints.

In specimens S_1_1 and E_1_1 (Figure 17), both zones of concentrated strain and zones where the values are close to zero are clearly visible. The near-zero values appear as a result of the delamination of the epoxy spot bond from the aluminum adherend. There is a lack of strain symmetry in specimen S_1_1, and the load is mainly transmitted through the upper rigid weld. It is likely that the central flexible bond and the lower stiff bond have partially failed, as can be observed in the force–displacement diagram in the form of upslopes. In specimen E_1_1, there is no doubt about the presence of strain symmetry. This is the stage at which the two elastic bonds are no longer carrying the load, as evidenced by the near circular blue strain fields. The load is carried by the middle part of the specimen, where the stiff bond is working together with the double-sided VHB tape.

Increasing the internal diameter of the elastic bond to 11 mm (specimen S_2_1, Figure 17), changes the load transfer arrangement. Concentrations occur at the location of the above-mentioned joint, while the upper stiff bond has been damaged. The strain field for specimen E_2_1 is more difficult to analyze, but it can be said with certainty that the load is mainly transferred through the central rigid bond.

The original strain distribution occurs for specimen S_3_1 (Figure 19). In this case, the load in the second stage is carried mainly by the central elastic joint. However, there is a lack of symmetry with respect to the vertical axis, as the upper right part of the lap is unloaded, while there are significant strains on the left side. This may indicate defects in the fabrication of the joint. For the E_3_1 sample, symmetry about the vertical axis is evident. It can also be seen that the lower elastic bond has delaminated, and the load is mainly transferred through the central part of the specimen, where there is a rigid bond.

For the S_4_1 specimen (Figure 20), strain concentrations can be seen in the central joint zone, which in this case is an elastic joint. In this case, it is likely that one of the rigid joints, closer to the edge, is no longer working. The situation is reversed for the E_4_1 specimen, where there are large deformations in the outer joint.

For the S_5_1 specimen (Figure 21), it is difficult to form conclusions, as there is a significant strain concentration almost throughout the lap area. On the other hand, it can be said with certainty that there is little deformation in the end zone of the lap in the E_5_1 specimen, so one of the elastic joints has already stopped working.

In conclusion, although it is not possible to directly observe the work of epoxy bonds, the Aramis DIC system, thanks to its high resolution, is able to record small changes in strains on the lap surface. This is highly advantageous not only for observing the work of individual joints, but also for the quality of their fabrication, which can be verified based on the symmetry of the strain fields with respect to the vertical axis.

## 4. Conclusions

In this study, uniaxial tensile tests were performed for 50 dual-adhesive samples (the first stage of the work) and 50 triple-adhesive samples (the second stage of the work). The goal of the first stage was to determine the two extremes in terms of the stiffness of the epoxy adhesives. From the 10 compositions analyzed, the most elastic was the 3M Scotch-Weld 2216 B/A Translucent adhesive, while the stiffest was the Epidian 53 (100 g) + “PAC” hardener (80 g). Both adhesives were used in the second stage of the work to makes triple-adhesive joints. In both stages of the work, a 1.6 mm thick VHB double-sided adhesive tape was also used in the lap. The following conclusions were drawn from the work:

1. The use of double-sided adhesive tape makes it possible to ensure a constant thickness of epoxy adhesive, simplifies assembly, and increases the aesthetics of the joint (no excess adhesive on the edges).
2. By changing the amount of PAC hardener added to the resin, an increase in bond strength can be achieved. The change is more evident for Epidian 53 (71% increase) than for Epidian 5 (31.5% increases).
3. By using double-sided adhesive tape and two types of epoxy adhesives with different stiffnesses, a three-stage joint operation was achieved.
4. The triple-adhesive joints provide significant scope for designing the characteristics of joints. Depending on the configuration, maximum force can be achieved in the first or second stage. The multi-stage nature makes these joints safe for use in the automotive industry.
5. By using double-sided adhesive tape, the energy required to damage the joint is several times greater than that for epoxy joints. This also gives the joint a reserve of strength.
6. By using the Aramis system, it was possible to indirectly determine the effort in the individual joints.

Dual and triple adhesive joints may have applications in civil engineering, such as in modular lightweight floor systems made of glass and steel [39]. The joint using double-sided adhesive tape would allow preassembly of the structure and ensure perfect geometry. In the second stage, adhesive joints would be made using epoxy adhesive inserted between the glass and steel surfaces bordered by double-sided adhesive tape. Such a solution will also ensure safety since the failure of the joint is multi-stage and prevents sudden disasters.

## Figures and Tables

**Table 1:** Data for epoxy compositions.

	Work Life (24 °C) [min]	Time to Handling Strength (24 °C) [h]	Curing Time (24 °C) [Days]
Distal Classic	120	24	7
Scotch-Weld 2216 B/A Translucent	120	12–16	30
Epidian 5 + PAC	180	6–8	7–14
Epidian 53 + PAC	180	6–8	7–14
Epidian 5 + Z1	35	6–8	7–14
Epidian 53 + Z1	35	6–8	7–14

**Table 2:** Relation between forces for “S” group.

Case Number	Relation between Forces	Internal Bond Diameter [mm]
1	F_1 > F_2	8, 11, 14
2	F_2 > F_1	16, 20
3	F_3 > F_2	8, 11
4	F_2 > F_3	14, 16, 20

**Table 3:** Relation between forces for “E” group.

Case Number	Relation between Forces	Internal Bond Diameter [mm]
1	F_1 > F_2	14, 16
2	F_2 > F_1	8, 11
3	F_3 > F_2	no such case
4	F_2 > F_3	8, 11, 14, 16

## Data Availability

The data presented in this study are available on request from the corresponding author.
